# Reaction and relaxation at surface hotspots: using molecular dynamics and the energy-grained master equation to describe diamond etching

**DOI:** 10.1098/rsta.2016.0206

**Published:** 2017-03-20

**Authors:** David R. Glowacki, W. J. Rodgers, Robin Shannon, Struan H. Robertson, Jeremy N. Harvey

**Affiliations:** 1 School of Chemistry, University of Bristol, Bristol BS8 1TS, UK; 2 Department of Computer Science, University of Bristol, Bristol BS8 1UB, UK; 3 Department of Mechanical Engineering, Stanford University, 452 Escondido Mall, Stanford, CA 94305, USA; 4 Dassault Systémes BIOVIA, 334 Cambridge Science Park, Cambridge CB4 0WN, UK; 5 Department of Chemistry, KU Leuven, Celestijnenlaan 200F, 3001 Heverlee, Belgium

**Keywords:** heterogeneous chemistry, reaction dynamics, molecular dynamics, statistical mechanics, chemical vapour deposition, diamond

## Abstract

The extent to which vibrational energy transfer dynamics can impact reaction outcomes beyond the gas phase remains an active research question. Molecular dynamics (MD) simulations are the method of choice for investigating such questions; however, they can be extremely expensive, and therefore it is worth developing cheaper models that are capable of furnishing reasonable results. This paper has two primary aims. First, we investigate the competition between energy relaxation and reaction at ‘hotspots’ that form on the surface of diamond during the chemical vapour deposition process. To explore this, we developed an efficient reactive potential energy surface by fitting an empirical valence bond model to higher-level *ab initio* electronic structure theory. We then ran 160 000 NVE trajectories on a large slab of diamond, and the results are in reasonable agreement with experiment: they suggest that energy dissipation from surface hotspots is complete within a few hundred femtoseconds, but that a small fraction of CH_3_ does in fact undergo dissociation prior to the onset of thermal equilibrium. Second, we developed and tested a general procedure to formulate and solve the energy-grained master equation (EGME) for surface chemistry problems. The procedure we outline splits the diamond slab into system and bath components, and then evaluates microcanonical transition-state theory rate coefficients in the configuration space of the system atoms. Energy transfer from the system to the bath is estimated using linear response theory from a single long MD trajectory, and used to parametrize an energy transfer function which can be input into the EGME. Despite the number of approximations involved, the surface EGME results are in reasonable agreement with the NVE MD simulations, but considerably cheaper. The results are encouraging, because they offer a computationally tractable strategy for investigating non-equilibrium reaction dynamics at surfaces for a broader range of systems.

This article is part of the themed issue ‘Theoretical and computational studies of non-equilibrium and non-statistical dynamics in the gas phase, in the condensed phase and at interfaces’.

## 1. Introduction

It has been known for some time that the dynamics of vibrational energy flow can play a pivotal role in determining the rates and pathways of unimolecular and bimolecular reactions in the gas phase [1–4]. The extent to which vibrational energy transfer dynamics can impact reaction outcomes in condensed phases remains an active research question. Over the past few years, a combination of molecular dynamics and master equation studies have provided examples of reaction outcomes in liquids which depend on energy transfer efficiencies [5–8]. Chemistry and reaction dynamics at the gas–surface interface [9–11] provide an interesting application domain for investigating the role that energy transfer plays in governing reaction outcomes because it offers an intermediate regime between the gas and liquid phases.

Developing predictive chemical models which are capable of providing detailed microscopic insight into gas–surface kinetics and dynamics is an important area within chemical dynamics research [11]. Not only do a wide range of important industrial, catalytic and environmental processes occur at the gas–surface interface [12,13], but reaction dynamics studies at the gas–surface interface open up a wide range of fundamental research questions [11,14–16], including the following. To what extent is it possible for gas–surface kinetics and dynamics to be described using statistical models, or are they inherently dynamical processes? What role does energy transfer at the gas–surface interface play in determining chemical reaction outcomes [17]? And how does charge transfer and breakdown of the Born–Oppenheimer approximation impact surface reactivity?

This paper focuses on the first two questions listed above—i.e. how well can statistical models describe reactivity at the gas–surface interface, and to what extent can we develop quantitative models of energy transfer? The energy transfer question is particularly interesting given that recent advances in both experimental and computational methods have revealed a range of gas–surface reaction systems which appear to exhibit some degree of mode selectivity [16]. For example, in dissociative chemisorption experiments of CH_2_D_2_ on a metal surface, Beck *et al*. showed that dissociation was more likely if two quanta of energy were placed into a single CH stretching motion than if slightly more energy was distributed across two CH stretching motions—i.e. the more highly excited bond breaks more readily [18]. From a fundamental perspective, these studies provide insight into the time scales for energy redistribution and dissipation at the gas–surface interface, and also help us to understand the appropriateness of the ergodicity assumption in gas–surface dynamics. This in turn enables us to determine whether the kinetics at surfaces is amenable to modelling using statistical rate theories (e.g. the energy-grained master equation (EGME)), or whether more detailed approaches based on explicit molecular dynamics are indeed required.

**Figure 1.**
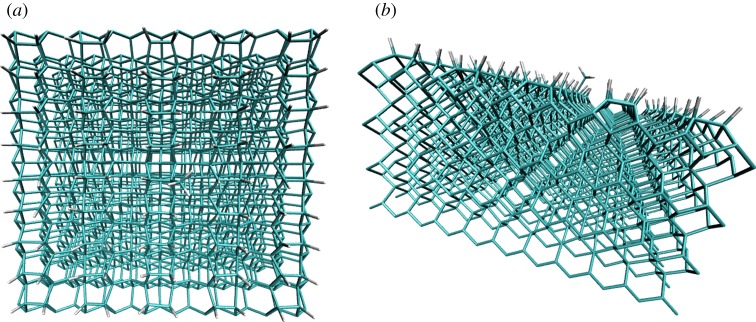
The C_1498_H_592_ diamond [100] slab that was modelled in this work. (*a*) A ‘top-down’ view, and (*b*) a ‘side-on’ view. The surface is hydrogen-capped except for a single –CH_3_ group, which is located near the centre of the slab, and easier to see in the ‘side-on’ view. (Online version in colour.)

In this paper, we investigate these questions by carrying out a detailed study of dissociation at the surface of a diamond slab shown in figure 1. Through comparisons between molecular dynamics (MD) simulations and EGME calculations, we show that dissociation at the diamond surface is in fact amenable to statistical treatments. There are two aspects which are key to the accuracy of the statistical approach outlined herein: (i) the system must be separated into ‘system’ and ‘bath’ components; and (ii) it must be possible to construct reasonably accurate models for energy transfer between the system and the bath. Our strategy for constructing an energy transfer model for surface reactions is straightforward: we have developed energy transfer functions which can be used within the EGME and which reasonably reproduce the time-dependent energy transfer profiles obtained in non-equilibrium MD simulations.

Chemical vapour deposition (CVD) is a common way of making diamond in the laboratory; however, there remains significant uncertainty regarding the microscopic chemical mechanisms that drive deposition and etching kinetics at diamond surfaces. The specific motivation of the method described in this paper is to provide further insight into the etching behaviour observed in CVD experiments. In particular, it has been shown that etching occurs in atomic H atmospheres. For example, net growth of single-crystalline diamond is observed under CVD conditions using an H_2_ microwave plasma in the presence of CH_4_, whereas in the absence of CH_4_, etching is observed [19]. Spontaneous thermal dissociation of C–C bonds has been suggested as a possible etching mechanism; however, computational studies suggest that the barriers for C–C dissociation are too high to explain the observed etching rates [20]. An alternative explanation invokes non-equilibrium kinetics at the diamond surface according to the following mechanism (where ‘S’ indicates the diamond surface):
R2a
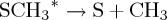

R2b
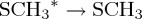


**Scheme 1.**
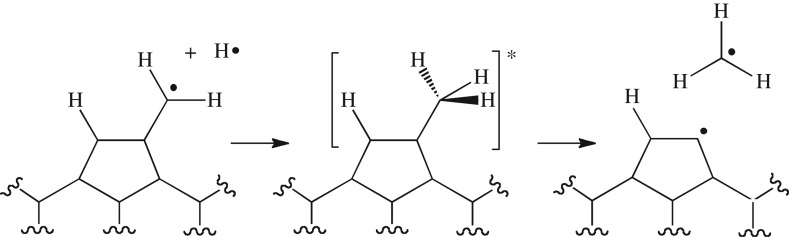
Mechanistic proposal for etching at the diamond surface under CVD conditions. Hydrogen atoms combine with dangling CH_2_ radicals at the diamond surface, creating a local ‘hotspot’ with approximately 100 kcal mol^−1^ excess energy, which is energetically sufficient to dissociate a CH_3_ radical.

The source of the internal energy indicated in (R2a) is H atoms, which combine with dangling CH_2_ radicals at the diamond surface, resulting in a local ‘hotspot’ containing approximately 100 kcal mol^–1^ excess energy. Within the nascent ‘hotspot’, it may be possible for CH_3_ dissociation (R2a) to occur before energy diffusion to the diamond bulk is complete (R2b), as illustrated in scheme 1.

In what follows, we have examined the mechanistic proposal shown in scheme 1 using both MD simulations and non-equilibrium statistical mechanics calculations. For the former, we carried out NVE MD simulations of a diamond slab. (In the microcanonical (NVE) ensemble, amount of substance (*N*), volume (*V*) and energy (*E*) are conserved.) C–C bond dissociation within these simulations was possible using an empirical valence bond (EVB) potential energy surface fitted to CASPT2 and CCSD(T) electronic structure theory. For the latter, we formulated and solved a stochastic EGME model that treats the competition between (i) CH_3_ dissociation (R2a) and (ii) energy diffusion out of the nascent hotspot into the bulk diamond (R2b). Energy transfer within the EGME model was treated using a function whose parameters were fitted to results obtained from the non-equilibrium MD simulations. As shown in what follows, the EGME (when compared to the results of MD simulations) is able to capture reasonably well the CH_3_ dissociation kinetics so long as the energy transfer time scales are appropriately modelled.

Because diamond is an efficient heat conductor, energy transfer from the system to the bulk is very fast. Nevertheless, both the MD simulations and the EGME suggest a small probability (approx. 0.1%) that CH_3_ dissociation occurs prior to the completion of energy diffusion. This dissociation probability yields a phenomenological etching rate in line with experiment. The extent to which EGME models can be applied beyond the gas phase remains an open question, with a critical issue being how to treat energy transfer beyond isolated binary collision conditions. The approach that we have used in this work—i.e. fitting energy transfer parameters based on non-equilibrium MD simulations—is one which has previously been used with success to describe energy transfer both in liquids [21] and also in the gas phase [22]. To the best of our knowledge, this study represents one of the first attempts to apply this methodology to reaction dynamics at the gas–surface interface.

The remainder of this paper is organized as follows. First, we describe the *ab initio* calculations we carried out in order to develop an accurate analytical –CH_3_ dissociative potential energy curve at the diamond surface. Second, we describe three different sets of MD simulations which we carried out in order to examine CH_3_ dissociation from the diamond surface. These included: (i) 160 000 non-equilibrium NVE trajectories in which the CH stretch of the surface methyl group was ‘plucked’ with the quantity of energy that would be available immediately following the H association step shown in scheme 1; (ii) thermal sampling along the –CH_3_ dissociation coordinate using the boxed molecular dynamics (BXD) method [23,24] in order to ascertain the free energy to dissociation and the corresponding thermal dissociation rate coefficient; and (iii) long NVE trajectories from which we backed out energy–energy correlation functions which we could use to fit the energy transfer parameters within our EGME model. Finally, we describe the EGME we formulated to model CH_3_ dissociation from the diamond surface, and show comparisons with the MD results.

## 2. *Ab initio* calculations for methyl dissociation

**Figure 2.**
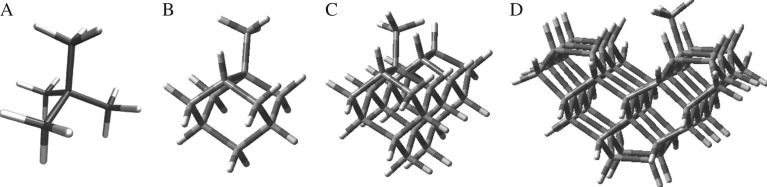
Model compounds A–D on which we carried out electronic structure calculations, chosen to mimic a diamond slab with a dangling –CH_3_ group.

A key component of the present study is the calculation of an accurate potential energy curve for the bond dissociation step (R2a). To avoid the expense of carrying out electronic structure calculations on a large diamond slab like that shown in figure 1, we instead carried out electronic structure calculations on a sequence of increasingly larger diamond proxy model compounds, the sequence of which is shown in figure 2. Using a range of methods, we initiated our model calculations by examining the dissociation of neopentane (model compound A in figure 2) to CH_3_ + C(CH_3_)_3_.

All density functional theory calculations reported in this work were obtained using the Gaussian 09 suite of programs [25], while all post-SCF (self-consistent field) and multi-reference calculations were obtained using the MOLPRO suite of programs [26]. According to the NIST webbook [27], the respective enthalpies of formation at 298 K of neopentane, methyl and t-butyl are –40.14 ± 0.15, 34.821 and 11.0 ± 0.7 kcal mol^–1^, suggesting a bond dissociation enthalpy of 86.0 ± 0.8 kcal mol^–1^. Calculations at the B3LYP/6-311G(d) level of theory predict that the dissociation energy at 0 K is 1.77 kcal mol^–1^ smaller than the dissociation enthalpy at 298 K, so we take the experimental value of the dissociation energy at 0 K to be 84.2 kcal mol^–1^. Table 1 shows the results of calculated bond energies using a variety of methods. It can be seen that the best calculated value at the CCSD(T)-F12 level of theory is 85.4 kcal mol^–1^, in very good agreement with the experimental value. The (H_3_C)_3_CCH_2_–H bond energy was also calculated at the B3LYP and CCSD(T) levels of theory, and the corresponding energies are shown in table 1.

**Table 1.** Calculated neopentane C–C (and C–H) bond dissociation energies (BDEs) in units of kcal mol^–1^.

	relaxed geometries^a^			rigid geometries^b^
	BDE_0_(C–C)^c^	BDE(C–H)^d^	^b^BDE(C–C)^d^	BDE_0_(C–C)^c^
B3LYP/6-311g*	84.5	98.0	76.1	102.4
B3LYP-D3/6-311G*	88.4	98.7	80.1	n.a.
MP2-F12/aug-cc-pVTZ^f^	99.0	100.5	90.6	116.7
CCSD-F12/aug-cc-pVTZ^f^	90.7	99.6	82.4	108.0
CCSD(T)-F12/aug-cc-pVTZ^f^	93.7	100.5	85.4	110.7
CASSCF/cc-pVTZ	75.7^c^	n.a	67.3^e^	91.0^e^
CASPT2/cc-pVTZ	90.3^c^	n.a.	82.0^e^	108.1^e^

^a^Energies obtained using the indicated method with the structures optimized using B3LYP.

^b^Energies obtained using the indicated method, and with fragment structures frozen to correspond with the B3LYP geometries at the neopentane minimum.

^c^Energies do not include zero-point energy corrections.

^d^Energies include B3LYP/6-311g* zero-point energy corrections.

^e^Dissociation limiting energies calculated for the supermolecule with *r*_CC_ = 10 Å.

^f^The cc-pVTZ basis set (not augmented) was used on hydrogen.

**Figure 3.**
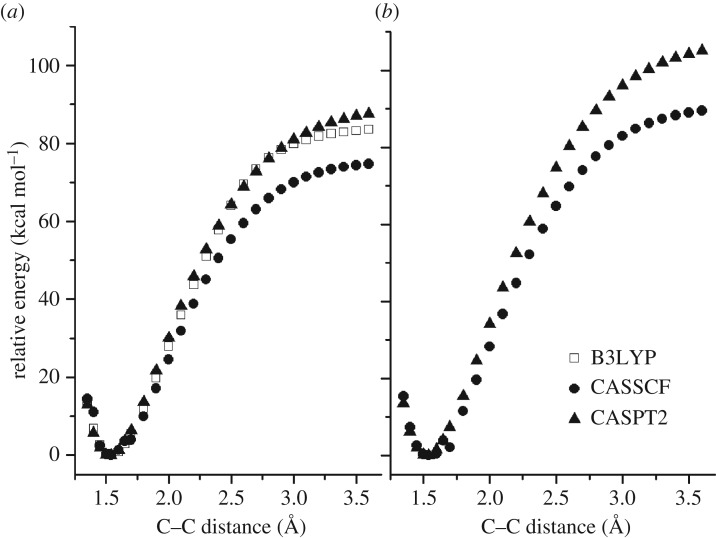
Scans along the –CH_3_ dissociation coordinate for neopentane, calculated at various levels of theory. Results obtained from (*a*) relaxed and (*b*) rigid scans.

In addition to the single point energy calculations given in table 1, we also carried out a set of scans along the C–C dissociation coordinate, plots of which are shown in figure 3. Because neopentane dissociation goes to a singlet diradical at large separations, an accurate treatment of the wave function along the dissociation coordinate requires the use of multi-reference methods. Our approach was to carry out both CASSCF(2,2)/cc-pVTZ and CASPT2(2,2)/cc-pVTZ energy calculations in which a two-electron, two-orbital active space was chosen to correspond to the singly occupied p orbital localized on each carbon during dissociation. Figure 3*a* shows CASPT2 and CASSCF results obtained at structures derived from relaxed B3LYP/6-311G(d) scans along the C–C reaction coordinate constrained to a C_3V_ symmetry. Table 1 shows the CASSCF and CASPT2 results at large separations (i.e. with *r*_CC_ = 10 Å), using the B3LYP optimized geometries. Note that the B3LYP calculations involved careful testing for a broken-symmetry unrestricted solution at each bond length, with the closed-shell solution found to be more stable until *r*_CC_ = 2.4 Å. The expectation value for the *S*^2^ operator for the Kohn–Sham orbitals then increases rapidly from 0.19 at *r*_CC_ = 2.6 Å to 0.92 at *r*_CC_ = 3.4 Å. It should also be noted that the CASSCF and CASPT2 curves show no barrier to dissociation in excess of the endothermicity.

The zero-point-corrected CASPT2 bond dissociation energy (BDE) of 82.0 kcal mol^–1^ is in good agreement with both experiment and the CCSD(T)-F12 BDE. Figure 3*b* shows the results of CASSCF and CASPT2 rigid scans along the C–C dissociation coordinate of neopentane. During these scans, C_3V_ symmetry was enforced, and the starting structure was the optimized B3LYP structure for neopentane. At large separations, the energies shown in figure 3*b* are larger than those shown in figure 3*a*, as the forming methyl and t-butyl radicals are not allowed to relax to their optimum structure. For example, the CASPT2 rigid-scan BDE (taken as the potential energy for *r*_CC_ = 10 Å) is 108.1 kcal mol^–1^, which is 17.8 kcal mol^–1^ larger than the energy obtained using B3LYP optimized geometries (i.e. a relaxed scan). Table 1 shows that the BDEs computed without fragment relaxation are all approximately 18 kcal mol^–1^ greater than the relaxed bond energies. The neopentane dissociation data in table 1 and figure 3 were used to fit an analytical potential energy surface model to accurately represent (R2a), which is discussed in further detail below.

In order to investigate the effect that the total system size had on the calculated –CH_3_ dissociation energy, we performed additional calculations on the incrementally larger diamond proxy models (structures B, C and D) in figure 2. Table 2 reports C–CH_3_ and CH_2_–H BDEs calculated for each of these structures using the B3LYP-D3 level of theory. The results in table 2 show that the smaller model B leads to an overestimate of the C–CH_3_ and –CH_2_–H BDEs at the diamond [100] surface, due to the lack of steric hindrance at the surface. In this respect, models C and D should be closer to the energies that we would expect at the surface of the diamond slab. The data in table 2 suggest that this is in fact the case, as the calculated bond energies appear to be converging to a limit as the system size increases. Note that the B3LYP bond energies are smaller than the B3LYP-D3 energies (especially for BDE(C–C)), owing to neglect of the attractive dispersive interactions between the methyl radical and the surface. A similar effect can also be seen for the C–C bond energy of neopentane in table 1. It is also worth noting that B3LYP-D3 underestimates the neopentane BDEs compared to both the experimental and the CCSD(T)-F12 values (both of which are in good agreement). Assuming that B3LYP-D3 makes a similar underestimate in the bond energy at the diamond [100] surface, our best estimates of the surface C─CH_3_ BDEs and CH_2_─H BDEs (based on the model D B3LYP-D3 value and the difference between the B3LYP-D3 and CCSD(T)-F12 neopentane values) are 86.6 and 93.6 kcal mol^−1^, respectively.

**Table 2.** Zero-point-corrected BDEs (kcal mol^–1^) obtained for the diamond proxy models B–D shown in figure 2, at the B3LYP/6-311G(d) and B3LYP-D3/6-311G(d) levels of theory. (Geometry optimization and zero-point energy corrections were calculated at the B3LYP/6-31G(d) level of theory.)

	B3LYP		B3LYP-D3	
	BDE(C–C)	BDE(C–H)	BDE(C–C)	BDE(C–H)
model B	87.1	96.4	90.8	97.1
model C	77.1	90.5	82.4	91.1
model D	73.6	90.8	81.3	91.8

## 3. Analytical potential energy surface for CH_3_ dissociation at the diamond surface

Non-equilibrium etching dynamics leading to dissociation of –CH_3_ from the diamond slab shown in figure 1 and scheme 1 turns out to be an extremely rare event. Accumulating the statistics required in order to assess the probability of this event required us to run 160 000 trajectories. This required an efficient dissociative potential energy surface (PES) with accurate energetics. *Ab initio* direct dynamics proved too computationally demanding for our purposes. Hybrid methods such as combined quantum mechanical and molecular mechanical (QM/MM) approaches might be tractable for optimizations and energy calculations [20,28–30], but are likewise too computationally demanding to carry out a large number of trajectory simulations. Instead, we chose to build on our previous work to date [8,31] using a parallel EVB [32] algorithm which we have implemented within the CHARMM molecular dynamics software [33].

**Figure 4.**
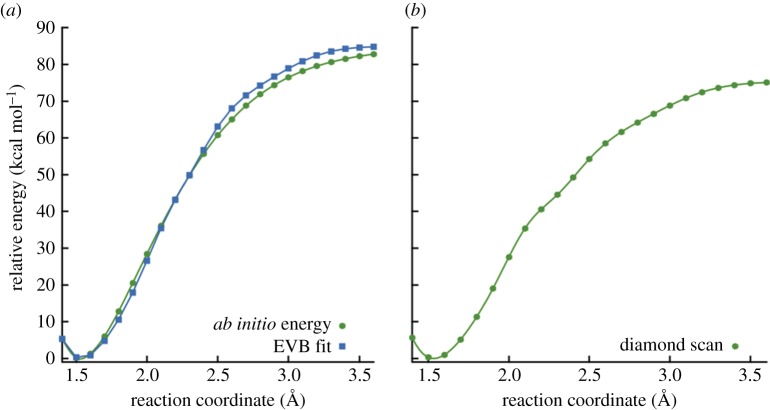
(*a*) The results of relaxed scans along the neopentane C–C bond, comparing the energies obtained from Δ*E*_*ab initio*_ and *λ*_0_ of the fitted EVB pseudo-Hamiltonian in equation (3.1). (*b*) The results of a relaxed scan along the C–C bond in the diamond slab shown in figure 1, using Hamiltonian parameters derived from the neopentane fit depicted in panel (*a*). (Online version in colour.)

Our preferred way to use the EVB method relies on fitting to higher-level electronic structure theory calculations. In this case, we chose to fit to the CASPT2 calculations shown in table 1 and figure 3, with a few caveats: it turns out that the CASPT2 method returns a relative energy at large C–C distances that is slightly smaller than our best estimate of the bond energy, discussed above. Furthermore, the CASPT2 energies along the dissociation curve cannot readily be corrected for zero-point energy. Accordingly, we scaled the CASPT2 BDEs along the figure 3 dissociation curve by *D*_0_(CCSD(T))/*D*_e_(CASPT2), where *D*_0_(CCSD(T)) is 85.4 kcal mol^–1^ (the zero-point-energy-corrected bond energy computed using CCSD(T)-F12) and *D*_e_(CASPT2) is 90.3 kcal mol^–1^ (the bond energy calculated using CASPT2 without zero-point correction). This yields a set of relative energies Δ*E*_*abinitio*_(*r_i_*), which are shown in figure 4*a*, and which were used to carry out the PES fitting described below.

Within the EVB approach, the potential energy for a given set of nuclear coordinates **q** is obtained as the lowest eigenvalue *λ*_0_ of a pseudo-Hamiltonian matrix,
3.1
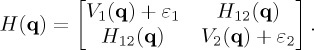

The diagonal elements *V_j_*(**q**) are molecular mechanics energies for the product and reactant structures. In this work, the diagonal elements of the matrix, *V*_1_(**q**) and *V*_2_(**q**), were obtained from the Merck molecular mechanics force field (MMFF94) [34] using appropriate bonding terms for the bonded and unbonded states, and modified to treat sp^2^-hybridized carbon radical centres like CH_3_.^1^ The role of the energy offsets 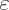
*_j_* (which are constant for all structures) is to correctly describe the electronic energy difference between reactant and product states. In the case that the respective coupling elements, *H*_12_(**q **= P) and *H*_12_(**q **= R), are close to zero near the product and reactant geometries, then the reaction energy is (*V*_2_(**q **= P) + 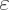
_2_) − (*V*_1_(**q **= R) + 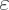
_1_). We represented off-diagonal elements *H*_12_(**q**) using a linear combination of *N* Gaussian functions (each of which depended on the C–C distance *r*) for the dissociating bond. The Gaussians had the form
3.2
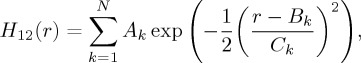

where *A_k_*, *B_k_* and *C_k_* are the respective amplitude, centre and width parameters for a particular Gaussian function. In the present case, the potential was fitted to the electronic structure data mentioned earlier using a combination of two Gaussian functions for the off-diagonal term (i.e. *N* = 2 in equation (3.2)). The fit was chosen to minimize a least-squares metric,
3.3
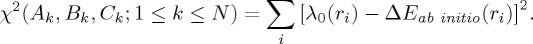

In this expression, *λ*_0_(*r*_*i*_) is the optimized value of the lowest eigenvalue of the matrix of equation (3.1), with the C–C distance *r* frozen to the same value *r*_*i*_ as that in the electronic structure calculations, but with all other structural parameters optimized. The value of the offset 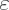
_1_ was chosen such that *V*_1_ + 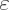
_1_ is equal to zero at the minimum of the potential energy curve, and such that *V*_2_ + 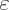
_2_ is equal to the *ab initio* dissociation energy at large *r*. The best-fitting parameters for each Gaussian are shown in table 3. The value of *H*_12_ is close to zero at distances *r* that are less than 1.54 Å and greater than 4.5 Å—i.e. the potential is described almost entirely by *V*_1_ in the reactant region and by *V*_2_ in the product region. A comparison between the fitted *λ*_0_ and Δ*E*_*abinitio*_ as a function of *r* is shown in figure 4*a*.

**Table 3.** Optimized parameters for the Gaussian functions making up *H*_12_ in equation (3.2).

Gaussian	*A* (amplitude)	*B* (centre)	*C* (width)
1	38.35	2.32	0.27
2	133.34	2.95	0.44

Owing to the difficulty of calculating *ab initio* values for the potential energy curve of CH_3_ dissociation from anything larger than structure D in figure 2, the EVB potential for CH_3_ dissociation was constructed as follows: The functional form and parameter values required to specify *H*_12_ were obtained from fits to *ab initio* energies along the neopentane dissociation curve (shown in figure 4*a*); the offset parameters 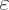
_1_ and 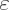
_2_ were specified so that *V*_1_ + 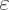
_1_ is equal to zero and *V*_2_ + 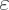
_2_ equals the –CH_3_ dissociation energy of structure D (see figure 2) at large *r*. A relaxed scan along the C–C reaction coordinate for the final parametrized diamond EVB model is shown in figure 4*b*. The fact that the neopentane fits in figure 4*a* can produce reasonable dissociation curves at the diamond surface (figure 4*b*) indicates that the fitted EVB parameters have some degree of transferability.

## 4. Molecular dynamics simulations of –CH_3_ dissociation at the diamond surface

### (a) NVE simulations

Having obtained an accurate and efficient –CH_3_ dissociation potential at the diamond surface, we investigated the relative rates of processes (R2a) and (R2b) using non-equilibrium NVE MD simulations. Here, ‘non-equilibrium’ refers to the fact that the initial conditions of each NVE trajectory were modified from coordinates and velocities selected from an NVT ensemble (details below), in order to emulate the nascent vibrationally excited SCH*_3_ species that we expect would be formed upon hydrogen atom addition to SCH_2_. (In the canonical (NVT) ensemble, amount of substance (*N*), volume (*V*) and temperature (*T*) are conserved.) MD trajectories were propagated in order to observe the fraction of trajectories in which prompt methyl formation was observed prior to the establishment of thermal equilibrium.

The procedure we used to carry out these MD simulations was as follows: A 1.6 µs (1 fs time step) NVT simulation of SCH_3_ was carried out using a Langevin thermostat with a friction coefficient of 10 ps^–1^ and heat bath of 1300 K (a typical temperature at which carbon etching is observed experimentally under [8] CVD growth conditions). Temperature equilibration was achieved within a few picoseconds, after which time coordinates and velocities from this trajectory were sampled every 10 ps (our tests showed this time to be a sufficiently long interval to ensure that the initial conditions were not significantly correlated to one another). This allowed us to generate initial conditions for 160 000 NVE simulations. Prior to running the NVE simulations, a non-equilibrium kinetic energy impulse was applied to one of the hydrogen atoms on the methyl group by modifying its NVT velocity in the direction of the CH bond, a local mode excitation strategy that we have used in previous work [8,35]. The magnitude of this velocity perturbation was chosen to correspond to a particular quantity of kinetic energy (*KE*), where *KE* = *E*_C–H_ + *E*_H_(*T*) + *E*_excess_. The C–H bond energy, *E*_C–H_, was specified to be 93.6 kcal mol^–1^ (our best guess for the diamond surface C–H BDE, discussed in relation to table 2). Energy *E*_H_(*T*) is the typical thermal translation energy for a hydrogen atom at 1300 K. And *E*_excess_ was an additional quantity of energy added to some of the runs in order to (i) increase the likelihood of a relatively rare –CH_3_ dissociation event, (ii) explore the sensitivity of the prompt –CH_3_ dissociation to initial energy and (iii) provide further data points for comparison with the non-equilibrium statistical mechanics approach described below. In the end, we ran three sets of NVE trajectories, each with a kinetic energy impulse *KE* = 108, 128 and 148 kcal mol^–1^. The non-equilibrium NVE trajectories were run for a total of 10 ps (1 fs time step). All of the dynamics were carried out using the leapfrog Verlet integration scheme, and the 858 atoms at the boundaries of the C_1498_H_592_ diamond slab model (figure 1) were frozen, leaving 1232 atoms that were free to move (for 3696 total degrees of freedom (d.f.)). As discussed below, the maximum excess energy added during our MD simulations was 148 kcal mol^−1^. In the limit that all of this energy is equipartitioned into the diamond slab on the time scale of a single MD simulation, this corresponds to an average of 0.04 kcal mol^−1^ per degree of freedom (i.e. 148.32 kcal mol^−1^/3696 d.f.), approximately 50 times smaller than *kT* (1.99 kcal mol^−1^) at 1000 K. This gave us confidence that our simulated diamond slab was large enough so as not to give rise to MD artefacts due to the system heating up as a result of adding non-equilibrium excess energy.

The trajectories were subsequently analysed in order to identify prompt –CH_3_ dissociation events. Any trajectory in which the dissociating C–C bond length exceeded 3 Å was counted as a dissociation event (inspection of dissociative trajectories showed that this was an effective point of no return). The results of the simulations are shown in table 4. Dissociation events were indeed observed over this energy range, which shows that (for a small number of trajectories) surface dissociation can occur prior to energy dissipation. In general, the number of dissociation events increases with the internal energy introduced via the non-equilibrium impulse applied to the C–H stretch. Despite the fact that these dissociation events are rare, there were enough events to count, which allowed us to derive approximate Poisson error estimates. The low probability of –CH_3_ dissociation arises from the fact that the excess energy in the C–H stretch is quickly dissipated into the bulk of the diamond slab (a point which is discussed in further detail below, with a typical time-dependent plot shown as electronic supplementary material, figure S1). When dissociation *did* take place, it necessarily occurred prior to dissipation of the initial C–H stretch energy to the bulk, and was therefore an extremely rapid process—i.e. within 100–200 fs.

**Table 4.** The fraction of prompt CH_3_ dissociation from the surface as a function of three different energetic impulses applied to the CH stretch. A Poisson distribution of rare events (*N*) was assumed, where the error is proportional to √*N*.

energy (kcal mol^−1^)	trajectories	CH_3_ dissociation events (*N*)	% dissociation
108	60 000	11	0.018 ± 0.006
128	60 000	65	0.065 ± 0.010
148	40 000	92	0.092 ± 0.016

### (b) Free energy sampling and thermal dissociation rate coefficient

The results in table 4 give the fraction of prompt dissociation events observed when the system has an energy which is initially in excess of the –CH_3_ BDE, and therefore provide insight into the *relative* rate of C–H energy dissipation versus CH_3_ dissociation. The energy dissipation rates in this system are extremely high. All of the observed dissociation events take place before the excess energy is dissipated and the system achieves thermal equilibrium, and are therefore very fast. In order to get a handle on how the time scales observed for the prompt dissociation events compare to the time scales observed for thermal dissociation, we calculated the absolute rate of the thermal CH_3_ dissociation (i.e. the dissociation rate coefficient at typical thermal energy distributions). We note that the time scales of the NVE simulations described above are too short to observe any thermal dissociation events. In order to back out the kinetics, we therefore used an accelerated free energy sampling technique called boxed molecular dynamics (BXD) [23,24,36]. The idea in BXD is to define a collective variable (or set of collective variables) which describes reaction progress, and then splice it into a set of ‘boxes’, which essentially correspond to hard-wall potential boundaries. A box is defined as a region of configuration space that lies between two boundaries; within any given box, a trajectory runs on the unbiased PES. If the trajectory crosses a particular box boundary, a velocity inversion operation [36] is performed to keep it within the specified box. BXD simulations are run by locking the system within a set of adjacent ‘boxes’, and then performing statistical analysis of the time spent in each box, and the relative number of hits at the boundaries that define the box. These quantities define box-to-box rate coefficients, which can then be used to calculate a potential of mean force, which is independent of the boundary locations. Choosing BXD boundaries is analogous to the process of specifying umbrellas (in umbrella sampling). A key difference is the fact that umbrella sampling requires two parameters per umbrella (location and force constant), whereas BXD requires only one parameter (location). While the latest BXD algorithms are able to adaptively determine the most computationally optimal box boundary locations [36] without requiring user specification, this capability was not available at the time the work described in this paper was carried out, and BXD boundaries were specified the ‘old-fashioned’ way (via user trial and error).

In order to calculate thermal dissociation rate coefficients, we performed BXD simulations from 700 K to 1300 K in 100 K intervals. The collective variable which we used to define progress along the –CH_3_ dissociation coordinate was the C–C bond distance. This coordinate was split into user-specified boxes of varying sizes, depending on the C–C bond distance: between 1.4 and 2 Å the boxes were 0.1 Å wide; between 2 and 3 Å the boxes were 0.2 Å wide; and between 3.3 and 4.0 Å the system was defined by a single box. Simulations were then run in each of these boxes, using an NVT ensemble with a Langevin heat bath (*T* = 1300 K, friction = 15 ps^–1^). Each simulation used a time step of 0.25 fs, and lasted for 187.5 ps. These settings ensured convergence of the BXD sampling within each box.

**Figure 5.**
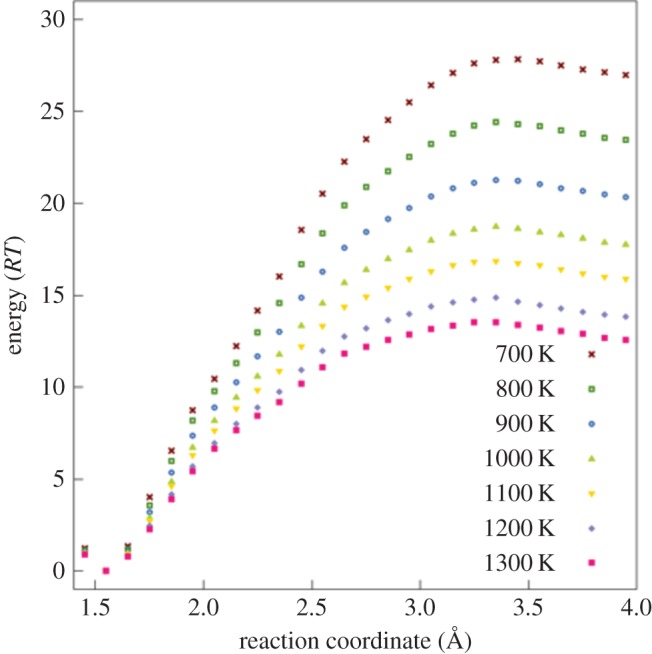
Temperature-dependent free energy surfaces of –CH_3_ dissociation at the diamond surface obtained using BXD at a range of temperatures. (Online version in colour.)

**Figure 6.**
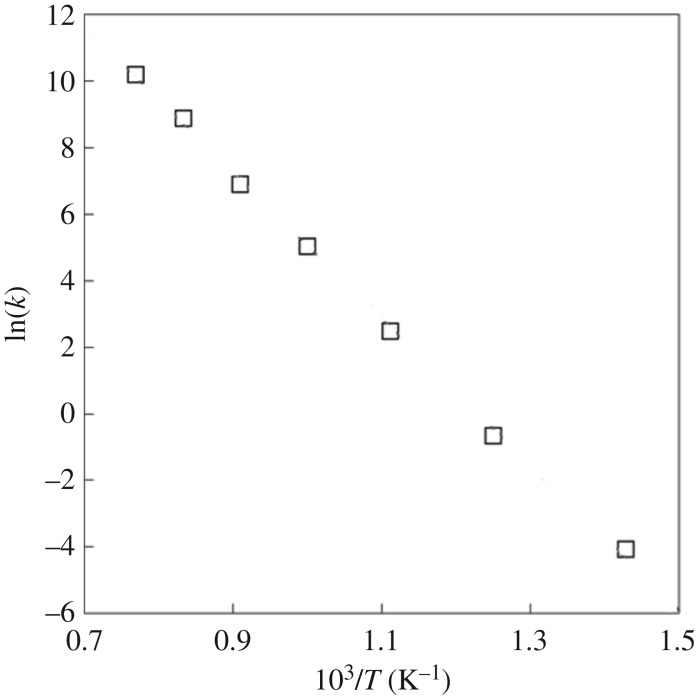
Arrhenius plot of table 5 rate constants. The points indicate the rate coefficients calculated from the Eyring equation using the BXD free energies of activation.

The free energy profiles calculated from BXD at each temperature are shown in figure 5. All of the free energy surfaces show the expected minimum at the C–C equilibrium bond length, then a steep increase, followed by a maximum at *r*_C–C_ ≈ 3.4 Å, followed by a decrease corresponding to the gain in entropy as the methyl radical dissociates. The maximum corresponds to the variational transition state for methyl loss. The origins of the variational transition-state barrier are primarily entropic (since there is no barrier on the underlying PES, see figure 4), and easiest to understand by thinking about the reverse process (i.e. CH_3_ association on the diamond surface): the barrier reflects the entropy penalty that CH_3_ pays for forming covalent bonds at the diamond surface. Table 5 gives Δ*G*_a_(*T*), the free energy of activation for CH_3_ dissociation at the diamond surface, calculated as the difference between the free energy at the variational transition state and the free energy minimum near the C–C equilibrium bond distance. Table 5 also shows the calculated thermal dissociation rate coefficients calculated using the Eyring equation, where *k*(*T*) = (*k*_B_*T*/*h*) exp(−Δ*G*_a_(*T*)/*RT*). Figure 6 shows an Arrhenius plot of *k*(*T*) plotted against 1/*T*.

**Table 5.** Free energy data obtained from BXD free energy profiles. The energies obtained are in units of *RT* and kcal mol^−1^; the reported rate constants were obtained by plugging the reported activation free energies into the Eyring equation.

temperature (K)	activation free energy (*RT*)	activation free energy (kcal mol^−1^)	rate constant (s^–1^)
700	27.84	38.72	1.2 × 10^1^
800	24.42	38.82	4.1 × 10^2^
900	21.28	38.06	1.1 × 10^4^
1000	18.72	37.20	1.5 × 10^5^
1100	16.86	36.85	1.1 × 10^6^
1200	14.88	35.48	8.6 × 10^6^
1300	13.55	35.00	3.5 × 10^7^

In a recent kinetic Monte Carlo modelling study of diamond growth under CVD conditions [37], it was found that good agreement with observed rates of growth could be found when assuming a rate constant for etching provided with an Eyring expression together with a temperature-independent free energy of activation of 200 kJ mol^−1^ (48 kcal mol^−1^). While this is larger than the values shown in table 5, it should be noted that this value in fact corresponds to the activation free energy for loss of an adsorbed ‘carbon’, which is typically present as a ─CH_2_ group inserted into a surface C–C bond. In the presence of excess H atoms in the diamond growth plasma, this can convert to a surface ─CH_3_ group, but this process is endothermic by approximately 80 kJ mol^−1^ (19 kcal mol^−1^) [20]. This suggests a free energy barrier for breaking the surface −CH_3_ bond of about 29 kcal mol^−1^, which is in satisfactory agreement with the BXD results in table 5, considering the uncertainty in the experimental analysis of the etching rate, and the inaccuracies of our EVB potential.

## 5. Master equation model of non-equilibrium dissociation

The results discussed above provide significant microscopic insight into the relative time scales of prompt (non-equilibrium) dissociation of CH_3_ at the diamond surface, and thermal (equilibrium) dissociation. However, the computational cost of the simulations outlined in the previous section is considerable: in addition to the thermal free energy sampling which we carried out using BXD, we also ran 160 000 non-equilibrium NVE trajectories. A key aim of the work described in the final part of this paper is to investigate whether it is possible to use a more computationally tractable approach for investigating non-equilibrium chemistry at surfaces, which avoids the need for large numbers of NVE trajectories, but which is able to provide results that are in line with the NVE trajectory predictions.

To this end, we formulated a non-equilibrium EGME model [38] to describe the competition between cooling and dissociation of the vibrationally excited SCH*_3_ at the diamond surface. All of the master equation and statistical mechanics calculations described below were carried out using MESMER [38], which is an open-source, cross-platform master equation code that we have been actively developing over the past few years. The master equation modelling approach outlined below builds on previous work in which we have carried out studies of non-equilibrium reaction dynamics in liquids [6]. The EGME is a computationally efficient framework typically used to describe the competition between relaxation and reaction dynamics for small molecules in the gas phase. It is essentially an energy-resolved system–bath model which describes the competing reaction and relaxation dynamics on a set of interlinked chemical intermediates. For this particular system, where we aim to model the competition between (R2a) and (R2b), the ansatz is as follows:
5.1
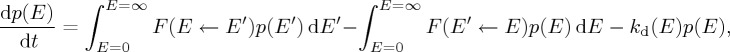

where *p*(*E*) is the population of SCH_3_ at a particular energy *E*, 
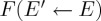
 is a function that describes the probability that the SCH_3_ population at energy *E′* is transferred (via bath interactions) to the SCH_3_ population at energy *E*, and *k*_d_(*E*) is the energy-resolved rate coefficient for dissociation of SCH_3_ at a particular energy to S + CH_3_, typically calculated as
5.2
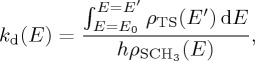

where *ρ*_SCH_3__(*E*) is the density of states of SCH_3_ and *ρ*_TS_(*E*) is the density of states of the CH_3_ dissociation transition state. Equation (5.2) works well when the reaction path in question has a well-defined PES barrier; however, it was not a good choice for the present case, where figure 4 shows that the PES does not have a well-defined barrier. As a result, we used an alternative approach, which allows a set of microcanonical rate coefficients *k*_d_(*E*) to be calculated from a modified Arrhenius rate expression for the corresponding canonical rate coefficients *k*(*β*), where *β =* (*κT*)^−1^. So long as *k*(*β*) can be represented by the modified Arrhenius expression,
5.3
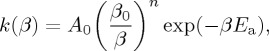

then it is possible to calculate *k*_d_(*E*) using an inverse Laplace transform (ILT) (where *L*^−1^[…] indicates an inverse Laplace transform of some argument […]), i.e. [38,39]
5.4
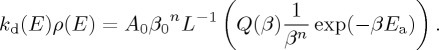

The *A_0_*, *B_0_*, *n* and *E*_a_ parameters in equations (5.3) and (5.4) were determined by fitting to the data in figure 6, and are discussed in further detail in the electronic supplementary material; in equation (5.4) *Q*(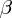) corresponds to the SCH_3_ partition function.

Equation (5.1) represents a set of coupled differential equations which is typically discretized into a set of contiguous intervals or ‘grains’ with energy spacing Δ*E* and written as a matrix equation:
5.5
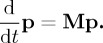

Diagonalizing the matrix **M** provides a solution for **p**, the population vector *p*(*E*), i.e.
5.6
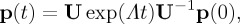

where **U** is the matrix of eigenvectors of **M**, and *Λ* a diagonal matrix of the corresponding eigenvalues. The EGME has advanced to the point that it is routinely able to provide near-quantitative predictions of gas-phase kinetics for reactive systems comprising multiple wells and transition states. Part of the reason for its success in the gas phase arises from the fact that the system/bath boundary is relatively well defined: the system comprises the reactive molecule of interest (which has available to it a range of reactive pathways), and the bath comprises third bodies which undergo collisions with the reactive system of interest. For example, the atmospheric bath is made up of N_2_ and O_2_, both of which can undergo elastic and inelastic collisions with the reacting species. Third-body collisions of this sort can be treated (often to a relatively high degree of accuracy) using standard functional forms that have their origins in isolated binary collision models, quantum mechanical scattering calculations and classical trajectory simulations.

**Figure 7.**
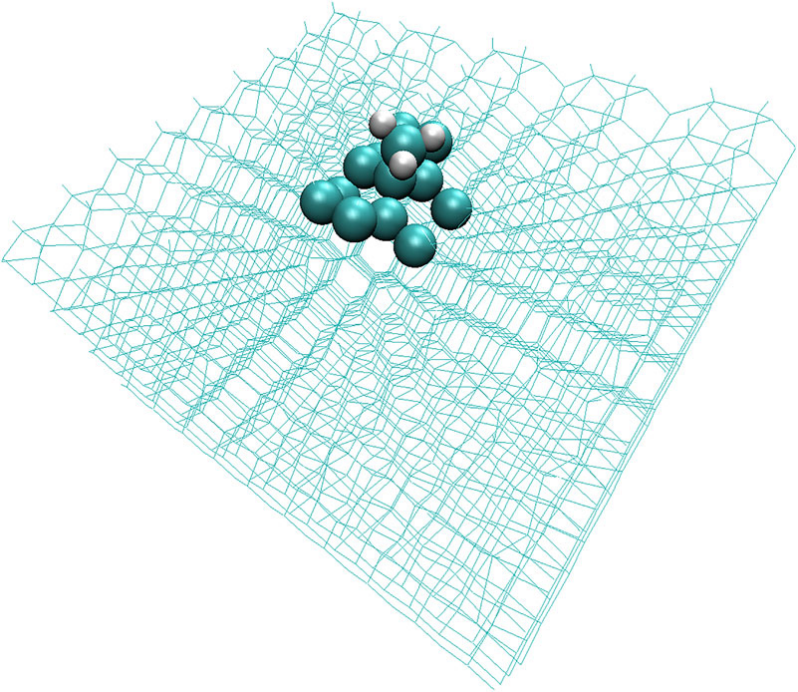
The 16 ‘system’ atoms at the surface of the diamond slab (including the pendent CH_3_) are shown as spheres, which are embedded in bath atoms (shown as a wireframe structure). (Online version in colour.)

Two important questions that arise in trying to apply system–bath models like the EGME to systems other than the gas phase are as follows: (i) How do we define the system/bath boundary? (ii) How do we treat energy transfer probabilities between the system and the bath? The approach that we have taken in this work is to use a simple distance-based criterion to define the system, which is shown in figure 7. We simply defined the reactive ‘system’ to comprise those atoms which are no more than four covalent bonds away from the C–H stretch to which the excess energy was initially added in the NVE simulations. Atoms which are more than four atoms away from the reactive system are treated as part of the bath. This definition led to a reactive system with 16 atoms.

**Figure 8.**
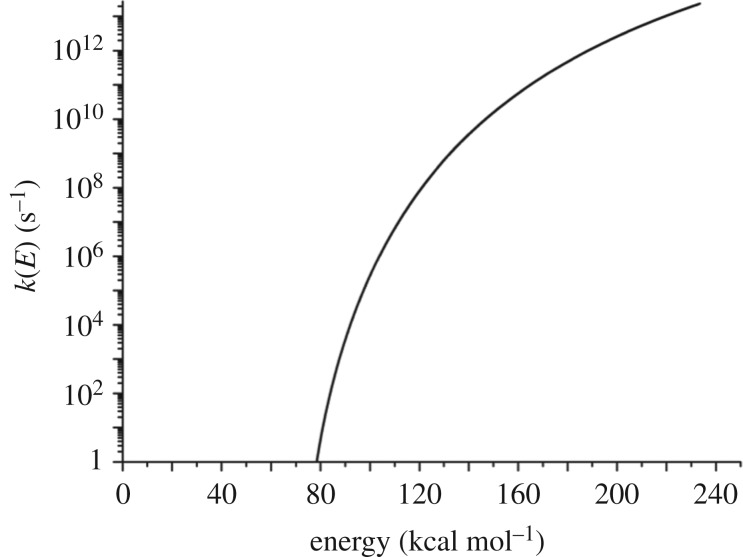
Value of *k*(*E*) for SCH_3_ dissociation to S + CH_3_ at the diamond surface, calculated via ILT as described in the text.

The microcanonical rate coefficients *k*_d_(*E*) required for solving equation (5.1) were obtained via equation (5.4) and require specification of the SCH_3_ density of states *ρ*(*E*). These were calculated in a locally modified version of CHARMM as follows: (i) a geometry optimization was carried out on the full diamond slab (system plus bath) shown in figure 4; (ii) the bath atoms in figure 7 were then frozen; (iii) a Hessian calculation was performed on the figure 7 system atoms; (iv) the system Hessian was then diagonalized, yielding a set of 3*n* vibrational frequencies (once the translational and rotational degrees of freedom were removed); and (v) energy-dependent densities of states for SCH_3_ *ρ*(*E*) were then calculated using the Beyer–Swinehart algorithm [38] within the harmonic oscillator approximation for the vibrational degrees of freedom only (i.e. rotational densities of states were set to zero). The *k*_d_(*E*) calculated using this procedure are shown in figure 8.

The energy transfer function *F*(*E* ← *E*′) required to solve equation (5.1) was obtained by fitting to results obtained from MD simulations, together with linear response theory. Specifically, a single 1 ns NVE simulation was performed (time step = 1 fs), using the full reactive EVB potential energy surface, and starting from an equilibrated structure generated during an NVT simulation at 1300 K. Atomic velocities were saved at each time step during the simulation, and the total kinetic energy of the atoms within the reactive subsystem was then computed from these velocities, in line with the strategy we have used successfully in previous work [8,21,31,40]. Specifically, the atomic kinetic energies were summed to construct a kinetic energy time series *KE*(*t*) for the ‘system’ atoms in figure 7. The average total energy within the system as a function of time 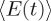 was calculated by the virial theorem as 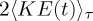, where the angled brackets indicate averages, and *τ* is a user-specified time window over which the averaging is carried out. This is a strategy that we have used successfully in a number of previous studies [5,8,31,40], which has given quantitative agreement with experiment, so long as *τ* is chosen to span several of the slowest vibrational periods within the set of ‘system’ atoms. For this system, we found that our results more or less converged so long as *τ* was greater than 100 fs. The 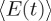 time series was then used to determine *C*(*t*), the time correlation function describing the total energy within the ‘system’ atoms,
5.7
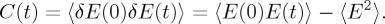


We note that, for this non-equilibrium, non-harmonic system, application of the virial theorem is not strictly appropriate. Ideally, we would instead compute the potential energy contribution from the subsystem, to yield 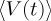. However, the energy expression used to propagate our MD simulations is not separable, so this is not straightforward. Nevertheless, except at very short time, the energy will be spread between many modes, so that roughly harmonic behaviour should be expected, as well as a reasonable balance between kinetic and potential energy. For this reason, we invoke the virial theorem, because it provides an approximate way to estimate the overall energy of the subsystem as a function of time. We note that an alternative approximation would have been to consider only the kinetic energy of the subsystem—this obviously decays on exactly the same time scale that we obtain here.

**Figure 9.**
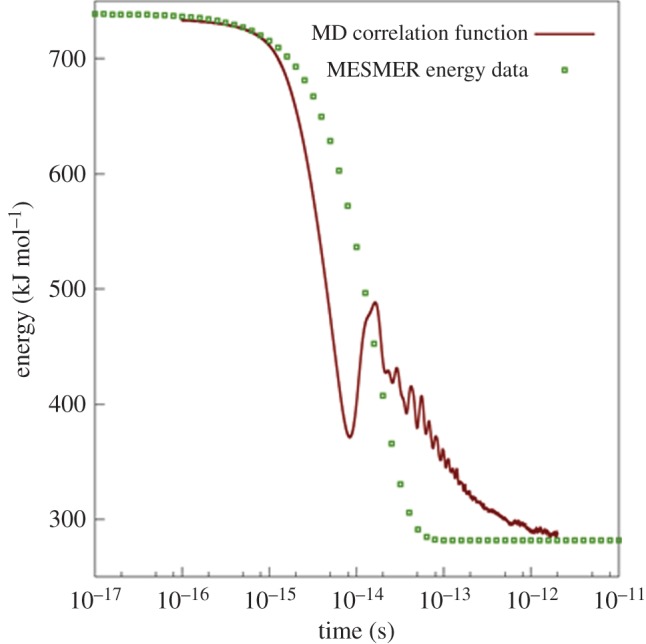
Comparison of energy correlation function obtained from MD (line) with that obtained through fitting to the EGME as described in the text (points). (Online version in colour.)

The resultant correlation function, shown in figure 9, provides information on the rate of energy transfer between the ‘system’ atoms in figure 7 and the ‘bath’ atoms. It shows that energy dissipation is complete within approximately 100 fs, and thus helps to explain the very short time scales observed for the prompt dissociation events in the previously described NVE simulations. It should be noted that the shape of the correlation function will be influenced to a certain extent, at short times, by the averaging over a period *τ*. With the MD correlation functions in hand, we then fit energy transfer functions *F*(*E* ← *E*′) for use in the EGME. These fits were carried out by varying the parameters in a Gaussian energy transfer function of the form
5.8
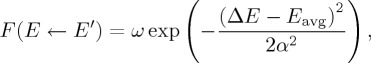

using a nonlinear least-squares algorithm to minimize the difference between (a) the MD correlation function in figure 9 and (b) the average time-dependent internal energy of SCH_3_ calculated in MESMER. In equation (5.8), the parameter definitions are as follows: Δ*E* = *E′* − *E*; *E*_avg_ is the most probable quantity of energy transferred; *α* is the width of the energy transfer distribution; and *ω* specifies the average frequency (s^−1^) at which energy transfer events occur. In the current simulations *E*_avg_, *α* and *ω* were determined to have values of 5.0 × 10^5^ cm^−1^, 4.5 × 10^5^ cm^−1^ and 5.9 × 10^14^ s^−1^, respectively. The approximate nature of the EGME model means that it is not possible to model the finer structure of the MD correlation functions, which arise from the detailed dynamics of coupled system–bath vibrations, and also from the ballistic nature of energy transfer at short times.

**Figure 10.**
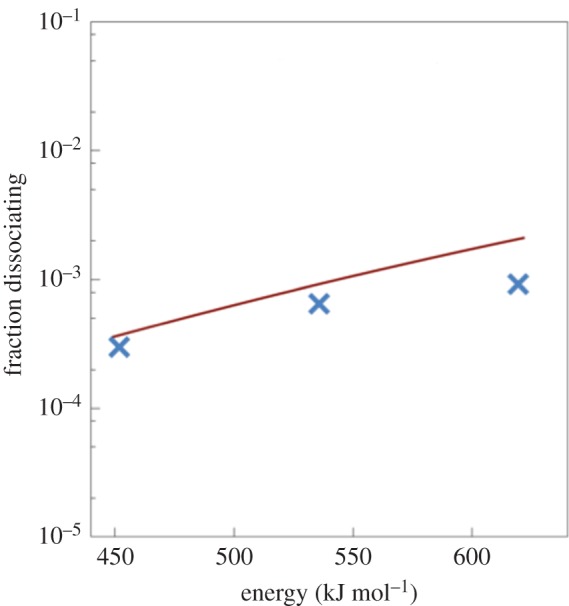
Comparison of the fraction of promptly dissociating CH_3_ calculated from the NVE MD simulations (crosses) and the EGME (solid line). (Online version in colour.)

Having calculated both *k*_d_(*E*) and *F*(*E *← *E*′), we were then able to solve equation (5.1), and evaluate the fraction of prompt dissociation events predicted by the EGME approach compared to the full NVE simulations. The results, shown in figure 10, were generated by changing the EGME initial conditions vector **p**(0) in equation (5.6) to correspond to delta-functions centred at the energies in table 4. The results in figure 10 indicate that the statistical EGME model does a reasonable job predicting the competition between reaction and relaxation within ‘system’ atoms. The agreement is particularly good at the lower energies, and slightly worse at higher energies. This is perhaps not an entirely unexpected result, since it is probably the case that non-statistical ballistic effects (which have a more accurate physical treatment in the MD simulations) are more important at higher energies.

The agreement between the MD and EGME results, while not perfect, is certainly surprising considering the number of approximations involved in formulating the EGME model, including: (i) the statistical approximation that is the basis for microcanonical transition-state theory, which neglects ballistic dynamical effects, and assumes that energy within the system is distributed ergodically; (ii) the fact that the boundary chosen to divide the system and the bath cuts across covalent bonds within the diamond slab; (iii) the fact that the energy transfer function *F*(*E *← *E*′) was fitted to a correlation function obtained from linear response theory using MD results from a single equilibrium NVE simulation; (iv) the approximate nature of the energy transfer function *F*(*E *← *E*′), which is unable to capture the finer dynamical structure of the correlation function obtained from MD simulations; and (v) the assumption that the rate at which the bath removes energy from the system is independent of where precisely that energy is located—i.e. the system is homogeneously coupled to the bath phonon modes.

## 6. Conclusion

In this work, we have carried out a detailed study of etching kinetics at the diamond surface under typical CVD conditions using MD simulations, and also a statistical EGME model. The aim of the work was twofold. First, we wanted to investigate the possibility that surface ‘hotspots’ might lead to prompt CH_3_ dissociation—i.e. dissociation that occurs prior to the onset of thermal equilibrium. To this end, we developed an efficient reactive PES by fitting an EVB model to higher-level *ab initio* electronic structure theory. Using this PES, we carried out 160 000 NVE trajectories, and observed that energy dissipation from surface hotspots on diamond is indeed very fast, but that a small fraction of CH_3_ does nevertheless undergo dissociation prior to the onset of thermal equilibrium. The results of the MD simulations are in reasonable agreement with experimentally observed etching rates.

Second, we wished to investigate the extent to which a significantly cheaper computational model—namely, a statistical EGME model—could capture the NVE trajectory results. To facilitate this aspect of the work, we outlined a general procedure for formulating and solving the EGME for surface chemistry. The idea is to split the system into system and bath components, and then carry out microcanonical transition-state theory in the configuration space of the system atoms. Energy transfer from the system to the bath is estimated using linear response theory from a single long MD trajectory, and fitted to an energy transfer function which can be input into the EGME.

Despite the number of approximations involved in formulating this model, the results obtained are in reasonable agreement with the results obtained from 160 000 NVE MD simulations, potentially offering a computationally tractable strategy for investigating non-equilibrium reaction dynamics at surfaces for a broader range of systems beyond the diamond system investigated here. The results obtained in this paper are encouraging insofar as they suggest that it is possible to treat complicated surface dynamics using models which are computationally much less expensive than full MD simulations; however, there are also a range of issues that need to be investigated in further detail moving forward. For example, it would be good to investigate the extent to which the EGME results depend on different methods for drawing a separation between the system and the bath degrees of freedom: How do the results change with differing system/bath boundaries? How sensitive are the –CH_3_ dissociation probabilities to system size—i.e. how large or small does the system need to be for the EGME results to deviate significantly from the MD results? The form of the energy transfer function also needs further study—e.g. do energy transfer functions that capture the finer structure observed in MD simulations improve the results? And how would such a model perform if it used energy transfer models which explicitly describe energy transfer probabilities in terms of the spectral coupling between the system vibrations and phonon vibrations within the bath? Finally, it would be good to obtain further insight into the energetic regimes over which linear response theory provides an accurate description of energy dissipation.

## Supplementary Material

Supplementary information

## Footnotes

1 We modified the MMFF protocol so that it did not throw errors if an sp^2^-hybridized carbon centre was bonded to three hydrogens. The CH bonding parameters in CH_3_ were taken to be identical to those used for the CH bonds in =CH_2_. Similarly, the improper torsion parameters for CH_3_ (which ensure a planar equilibrium geometry) were taken to be identical to those for =CH_2_.
