# Supplementary material for: Reaction and relaxation at surface hotspots: using molecular dynamics and the energy-grained master equation to describe diamond etching
Source: Philos Trans A Math Phys Eng Sci. 2017 Mar 20;375(2092):20160206. doi: 10.1098/rsta.2016.0206 (PMC5360904; doi:10.1098/rsta.2016.0206)
Supplement: Supplementary information [file rsta20160206supp1.docx]

Supplementary information

**Reaction and Relaxation at surface hotspots: using molecular dynamics and the energy-grained master equation to describe diamond etching**

David R. Glowacki,^1,2^* W. J. Rodgers,^1^ Robin Shannon,^1,3^ Struan H. Robertson,^4^

Jeremy N. Harvey^5^

*^1^School of Chemistry, University of Bristol, Bristol, BS8 1TS, UK; ^2^Department of Computer Science, University of Bristol, Bristol BS8 1UB, UK; ^3^Department of Mechanical Engineering, Stanford University, 452 Escondido Mall, Stanford, CA 94305, USA; ^4^Dassault Systémes, Biovia, 334 Cambridge Science Park, Cambridge CB4 0WN, UK; ^5^Department of Chemistry, KU Leuven, Celestijnenlaan 200F, B-3001 Heverlee, Belgium*

*glowacki@bristol.ac.uk

This supplementary Information includes the following:

1. Fig S1, showing a typical Plot of total energy vs. time in the C–H bond which was plucked at time zero in the non-equilibrium trajectories
2. The MESMER input file used to carry out the EGME modeling described within the text. MESMER is available online at http://sourceforge.net/projects/mesmer/


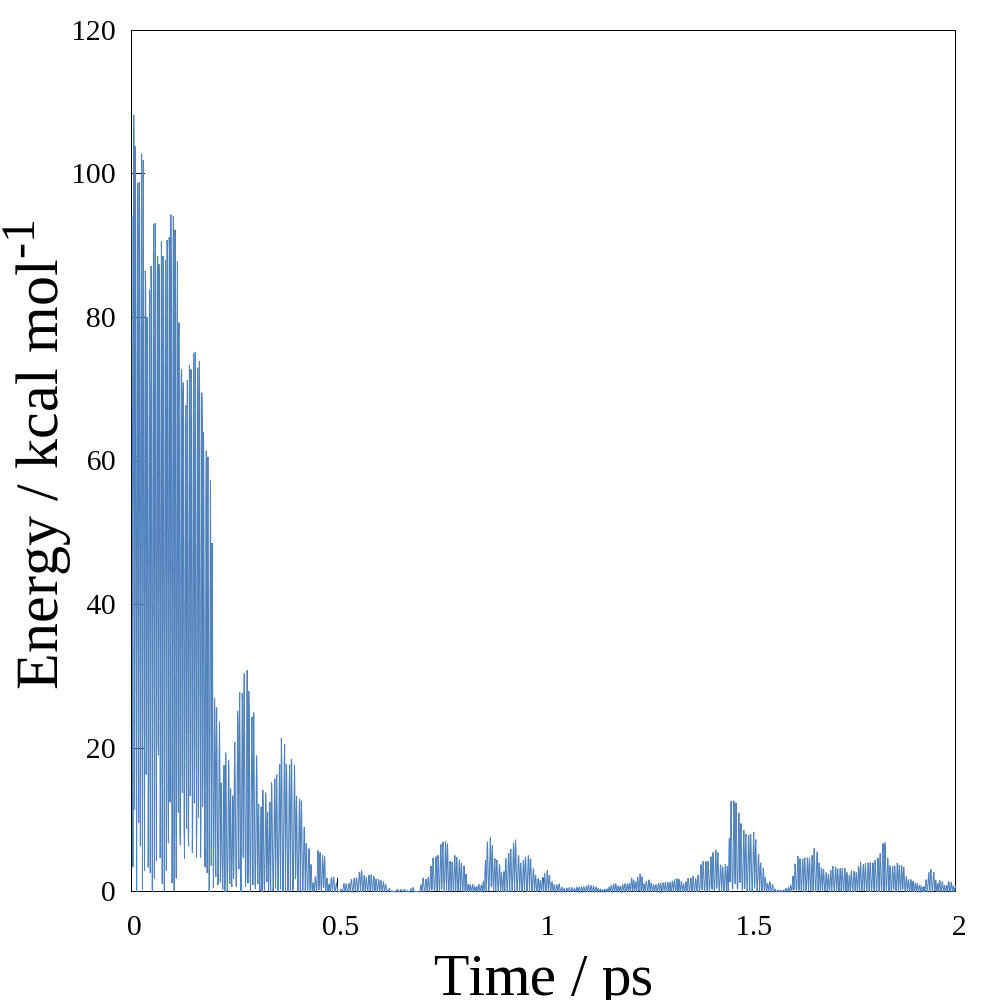


Figure S1: Typical Plot of the energy in the ‘plucked’ C–H bond as a function of time

Mesmer Input File for EGME modelling

Note on the Inverse Laplace Transform (ILT) parameters: The ILT parameters used in the MESMER input below were obtained by fitting the data points in Fig 6 of the main text to Eq (6) in the main text. In carrying out this fit, it is important to mention one key difference between Eq (6) and the standard Arrhenius expression [i.e., ln(*k*) = ln(*A*) – (*E_a_*/*R*)(1/*T*)]. In the standard Arrhenius expression, *E_a_* is a purely phenomenological observable corresponding to the free energy of activation. However, the *k*(*E*)s one obtains from Eq (6) are most accurate when *E_a_* has a value which is close to the actual 0K activation barrier. The expression in Eq (6) affords a degree of flexibility that is not available in the standard Arrhenius expression owing to the fact that it includes the exponential parameter *n*. During our fits of the Fig 6 data using Eq (6), we therefore constrained the value of *E_a_* to 315 kJ mol^-1^ (~75 kcal mol^-1^), which is effectively the value of the CH_3_ BDEs calculated in the main text (see Table 2 and Fig 4b).

<?xml version="1.0" encoding="utf-8" ?>

<?xml-stylesheet type='text/xsl' href='../../mesmer2.xsl' media='other'?>

<?xml-stylesheet type='text/xsl' href='../../mesmer1.xsl' media='screen'?>

<me:mesmer xmlns="http://www.xml-cml.org/schema" xmlns:me="http://www.chem.leeds.ac.uk/mesmer">

<title>Diamond</title>

<moleculeList>

<molecule id="CH3">

<propertyList>

<property dictRef="me:ZPE">

<scalar units="kJ/mol">0.0</scalar>

</property>

<property dictRef="me:MW">

<scalar units="amu">15.0</scalar>

</property>

</propertyList>

<me:DOSCMethod>ClassicalRotors</me:DOSCMethod>

</molecule>

<molecule id="activeSpaceRadical">

<propertyList>

<property dictRef="me:ZPE">

<scalar units="kJ/mol">315.0</scalar>

</property>

<property dictRef="me:MW">

<scalar units="amu">156.0</scalar>

</property>

</propertyList>

<me:DOSCMethod>ClassicalRotors</me:DOSCMethod>

</molecule>

<molecule id="5AtomActiveSpace">

<propertyList>

<property dictRef="me:ZPE">

<scalar units="kJ/mol">0.0</scalar>

</property>

<property dictRef="me:symmetryNumber">

<scalar>1</scalar>

</property>

<property dictRef="me:vibFreqs">

<array units="cm-1">

107.255912

342.337739

361.562987

404.568852

496.669203

562.973352

643.165972

650.850447

707.07495

712.855143

723.693015

741.443389

775.571092

788.281668

841.1611

862.890901

883.461487

924.645472

928.147584

957.404406

957.589713

967.221202

972.694228

986.210941

1000.037423

1036.7015

1043.665076

1065.133472

1075.97649

1093.99322

1119.001941

1131.226761

1156.503446

1164.343463

1174.897912

1176.356843

1216.76092

1224.840072

1230.527312

1244.628961

1259.777221

1270.039708

1310.758585

1330.387608

1435.391102

1514.271399

1542.564995

2885.256444

2943.600679

2985.384695

3055.284743

</array>

</property>

<!--the following properties, MW, epsilon, sigma, and deltaEDown, are used only for LJ collisional energy transfer, so not too important here

except as adjustable parameters -->

<property dictRef="me:MW">

<scalar units="amu">171</scalar>

</property>

<property dictRef="me:epsilon">

<scalar>250.0</scalar>

</property>

<property dictRef="me:sigma">

<scalar>5.0</scalar>

</property>

<!--<property dictRef="me:deltaEDown">

<scalar units="cm-1">50000</scalar>

</property>-->

<property dictRef="me:gaussianCenter">

<scalar units="cm-1">45000</scalar>

</property>

<property dictRef="me:gaussianWidth">

<scalar units="cm-1">6000</scalar>

</property>

<property dictRef="me:spinMultiplicity">

<scalar>1</scalar>

</property>

</propertyList>

<me:energyTransferModel>gaussian</me:energyTransferModel>

<me:DOSCMethod>ClassicalRotors</me:DOSCMethod>

</molecule>

<molecule id="N2">

<atom elementType="N2" />

<propertyList>

<property dictRef="me:epsilon">

<scalar>48.0</scalar>

</property>

<property dictRef="me:sigma">

<scalar>3.90</scalar>

</property>

<property dictRef="me:MW">

<scalar units="amu">28.0</scalar>

</property>

</propertyList>

</molecule>

</moleculeList>

<reactionList>

<reaction id="R1">

<reactant>

<molecule ref="5AtomActiveSpace" me:type="modelled" />

</reactant>

<product>

<molecule ref="CH3" me:type="sink" />

</product>

<product>

<molecule ref="activeSpaceRadical" me:type="sink" />

</product>

<me:MCRCMethod>MesmerILT</me:MCRCMethod>

<me:preExponential>4.0e19</me:preExponential>

<me:activationEnergy units="kJ/mol" reverse="false">315.0</me:activationEnergy>

<me:TInfinity>1100.0</me:TInfinity>

<me:nInfinity>0.01</me:nInfinity>

</reaction>

</reactionList>

<me:conditions>

<me:bathGas>N2</me:bathGas>

<me:PTs>

<!--<me:PTpair me:units="Torr" me:P="2000000000" me:T="1300.0" />-->

<me:PTpair me:units="Torr" me:P="150000000" me:T="1300.0" />

</me:PTs>

<me:InitialPopulation>

<molecule ref="5AtomActiveSpace" me:population="1.0" me:grain="211" />

<!--<molecule ref="5AtomActiveSpace" me:population="1.0" />-->

</me:InitialPopulation>-->

</me:conditions>

<me:modelParameters>

<!--Specify grain size directly...-->

<me:grainSize units="cm-1">350</me:grainSize>

<!--...or by the total number of grains

<me:numberOfGrains> 500 </me:numberOfGrains>-->

<!--Specify increased energy range

<me:maxTemperature>6000</me:maxTemperature>-->

<me:energyAboveTheTopHill>60.</me:energyAboveTheTopHill>

</me:modelParameters>

<me:control>

<me:testDOS />

<me:testMicroRates />

<!--<me:printGrainDOS />-->

<!--<me:printCellDOS />-->

<!--<me:printReactionOperatorColumnSums />-->

<me:printGrainkfE />

<me:printGrainedSpeciesProfile/>

<!--<me:printGrainBoltzmann />-->

<me:printGrainkbE />

<me:printSpeciesProfile />

<me:testRateConstants />

<me:shortestTimeOfInterest>1.0e-17</me:shortestTimeOfInterest>

<me:eigenvalues>all</me:eigenvalues>

<me:printReactionOperatorColumnSums />

<me:useDOSweighedDownWardTransition />

<me:printCollisionOperatorLevel> 1 </me:printCollisionOperatorLevel>

</me:control>

</me:mesmer>
